# USDA Nutrition Evidence Systematic Review’s collaborative approach for conducting systematic reviews: Promoting diversity of expertise while managing potential conflicts of interest

**DOI:** 10.3389/fnut.2023.1112868

**Published:** 2023-02-23

**Authors:** Julie Obbagy, Ramkripa Raghavan, Natasha Chong Cole, Laural K. English, Molly Higgins, Joanne M. Spahn, Charlotte L. Bahnfleth, Emily Callahan, Amanda Fultz, Julia H. Kim, Brittany J. Kingshipp, Julie E. H. Nevins, Sara R. Scinto-Madonich, Allison Webster, Eve Stoody

**Affiliations:** ^1^Nutrition Evidence Systematic Review (NESR) Branch, Nutrition Guidance and Analysis Division (NGAD), Center for Nutrition Policy and Promotion (CNPP), Food and Nutrition Service (FNS), U.S. Department of Agriculture (USDA), Alexandria, VA, United States; ^2^Nutrition Guidance and Analysis Division (NGAD), Center for Nutrition Policy and Promotion (CNPP), Food and Nutrition Service (FNS), U.S. Department of Agriculture (USDA), Alexandria, VA, United States

**Keywords:** dietary guidelines, nutrition evidence systematic review, systematic review, public health, nutrition, NESR, conflict of interest, systematic review methods

## Abstract

U.S. Department of Agriculture’s (USDA) Nutrition Evidence Systematic Review (NESR) Branch develops food-and nutrition-related systematic reviews and other evidence synthesis products. NESR has established itself as a key resource for the Federal government when making evidence-informed decisions related to public health nutrition, such as the development of the *Dietary Guidelines for Americans*. NESR’s systematic review methodology is rigorous, protocol-driven, and highly collaborative. NESR’s systematic reviews examine the complex interplay between diet and health with input and support from various collaborators, including Federal stakeholders, expert groups, and public stakeholders. Implementing NESR’s rigorous methodology ensures that the appropriate steps are taken to minimize conflict of interest, producing systematic reviews that are high-quality, trustworthy, and useful to end users who make decisions based on their findings. This article describes how NESR’s systematic review process leverages a diversity of expertise and experience, while managing potential conflicts of interest. It describes the groups who collaborate to conduct NESR systematic reviews, their expertise, and why their involvement is critical for ensuring the rigor and utility of NESR’s work.

## Introduction

In 2008, the U.S. Department of Agriculture’s (USDA), Food and Nutrition Service, Center for Nutrition Policy and Promotion (CNPP) launched the Nutrition Evidence Systematic Review (NESR) Branch to develop food-and nutrition-related systematic reviews and other evidence synthesis products. NESR was created to support CNPP’s mission of improving the health of Americans by developing and promoting dietary guidance that links scientific research to the nutrition needs of consumers. Public health nutrition decision-making is strengthened when informed by scientific evidence – and NESR has established itself as a key resource for the Federal government when making evidence-informed decisions related to public health nutrition, such as the development of the *Dietary Guidelines for Americans*.

NESR systematic review projects uphold the Data Quality Act (1), which mandates that Federal agencies ensure the quality, objectivity, utility, and integrity of the information used to create Federal guidance. In addition, the NESR Branch embodies the principles of USDA’s Scientific Integrity Policy, which specifies that USDA employees who use scientific information to support policy-and decision-making are responsible for ensuring the quality, accuracy, and transparency of that information, and should do so without political or inappropriate influence (2). Further, NESR’s approach to reviewing nutrition science embraces five values identified by a National Academies committee as being critical for the development of credible and trustworthy guidelines: transparency, management of biases and conflicts of interest, diversity of expertise and experience, a deliberative process, and the adoption of state-of-the-art processes and methods (3).

NESR’s systematic review methodology is rigorous, protocol-driven, and highly collaborative. NESR’s systematic reviews examine the complex interplay between diet and health with input and support from various collaborators, including Federal stakeholders, expert groups, and public stakeholders. Implementing NESR’s rigorous methodology ensures that the appropriate steps are taken to minimize bias, producing systematic reviews that are high-quality, trustworthy, and useful to end users who make decisions based on their findings. To help meet goals for transparency and reproducibility, complete documentation of NESR’s work is made accessible to the public on the NESR website.^1^

This article serves as an opportunity to describe how NESR’s systematic review process leverages a diversity of expertise and experience, while managing potential conflicts of interest (COI). It describes the groups who collaborate to conduct NESR systematic reviews, their expertise, and why their involvement is critical for ensuring the rigor and utility of NESR’s work.

## The Nutrition Evidence Systematic Review team

The NESR Branch consists of a team of career Federal scientists, including analysts and librarians. Given that the NESR branch is housed within the USDA, the team adheres to USDA’s Scientific Integrity Policy and other Agency and Federal government standards of ethical conduct (2).

NESR analysts are scientists with doctoral (PhD, DrPH) or master’s (MS, MPH) degrees in nutritional science or related fields such as public health, biochemistry, or biology. Some also have a registered dietitian (RD) or registered dietitian nutritionist (RDN) credential. NESR analysts have demonstrated expertise in conducting research on a range of diet and health-related topics (4). They also have extensive training and expertise in systematic review and evidence synthesis methodology (e.g., risk of bias assessment, grading the strength of evidence, meta-analysis), technology (e.g., software for literature screening, data extraction, reference management, and statistical analyses), and meeting facilitation. In addition, they have experience with science communication, including publishing peer-reviewed articles, presenting to diverse audiences, developing scientific reports, writing plain language summaries, and curating website content.

NESR librarians are information specialists with master’s degrees in library and information science. NESR librarians have extensive training and expertise in methodology and technology (e.g., software for literature screening, reference management used for developing, implementing, and documenting literature searches) for a range of diet and health-related topics. Specifically, they also have knowledge of bibliographic databases (e.g., PubMed/MEDLINE, Cochrane, Embase, and CINAHL), including search terms appropriate for each database, and search refinements, such as search filters.

## How NESR works with collaborators to conducts its systematic reviews

### Collaborators

**Figure 1:**
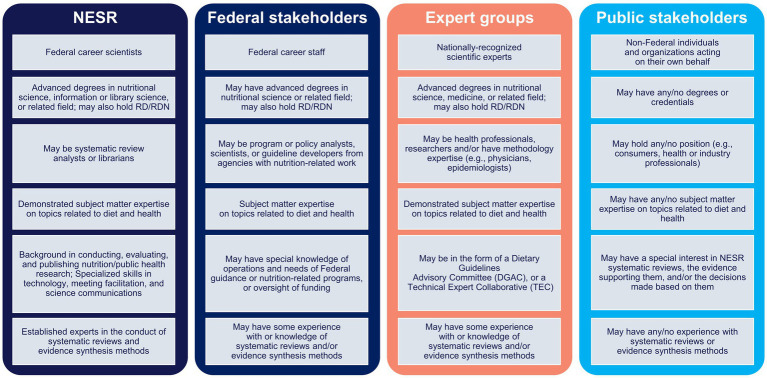
NESR and Collaborator positions, credentials, roles, expertise and special skills.

NESR systematic reviews are conducted with input and support from various collaborators, including Federal stakeholders, expert groups, and public stakeholders. Having the input and support of diverse collaborators, who each provide informed and unique perspectives, helps maintain the rigor, integrity, and trustworthiness of NESR systematic reviews as well as the overall process. NESR’s systematic review process and methodology leverage collaborators’ expertise, while clearly delineating roles and responsibilities, and managing potential COI throughout. Figure 1 overviews the expertise of NESR and its various collaborators. Below is a detailed description of the collaborators involved in the NESR systematic review process.

#### Federal stakeholders

Federal stakeholders encompass a broad range of Federal career staff from agencies with nutrition-related work in one or more of their mission areas, such as the USDA, U.S. Department of Health and Human Services (HHS), U.S. Department of Veterans Affairs, U.S. Environmental Protection Agency, U.S. Department of Defense, and the U.S. Agency for International Development. Federal stakeholders may be scientists, nutritionists, program-and policy analysts, or guideline developers who have an interest in NESR systematic reviews and/or the evidence supporting them. Federal stakeholders may have advanced degrees in nutritional science or a related field, subject matter expertise in topics related to diet and health, knowledge of the operations and needs of Federal guidance or nutrition-related programs, experience with systematic reviews or evidence synthesis methods, or oversight of research funding. As employees of the U.S. government, Federal stakeholders must adhere to relevant scientific integrity policies within their agency, as well as other statutes, regulations, and agency policies governing ethical conduct (5–15).

#### Expert groups

Expert groups consist of nationally recognized scientific experts with demonstrated subject matter expertise on the topic (s) addressed in the NESR systematic review project. These scientific experts have advanced degrees in nutritional science, medicine, and/or a closely related field. Experts may be health professionals (e.g., physicians, registered dietitians, and public health practitioners), researchers (e.g., trialists, epidemiologists, and food scientists), and/or have specific methodology expertise (e.g., data analysis, systematic review, meta-analysis, or food pattern modeling). Expert group members are selected based on their scientific expertise, while also ensuring that membership is balanced and diverse in terms of points of view, expertise, experience, education, and institutional affiliation, as well as race and ethnicity, gender, and geographic location. They may be Federal employees or external to the Federal government. Potential COI are also assessed and taken into consideration when forming expert groups to determine whether anything could potentially bias the technical contributions of an individual or give the appearance of bias. If such potential conflicts are found, the individual expert may not be invited to participate, or, the individual may participate, but their conflicts are managed by the NESR team. Management of COI involves ensuring that the input of *all* expert group members is used to make decisions, such that a range of diverse viewpoints and subject matter expertise are considered and balanced when evaluating the evidence.

NESR collaborates with two types of expert groups, a Technical Expert Collaborative (TEC), or a Federal Advisory Committee (FAC) known as the Dietary Guidelines Advisory Committee.

##### Technical expert collaborative

A TEC is a group of approximately six to eight subject matter experts convened by NESR to conduct one or more systematic reviews on a specific topic. TEC members are identified by NESR and other Federal stakeholders, may be internal or external to the Federal government, and serve without pay for a period of 1–2 years, depending on the size and scope of the project. TEC members disclose potential COI prior to their appointment and periodically during the project, which are reviewed by the USDA Office of Ethics to ensure members have no financial COI that would prohibit serving on the TEC or that would require management by the NESR team. TEC members collaborate with NESR to conduct systematic reviews (16–18) that describe the state of the science on a particular topic and question, but because a TEC is not a FAC, it does not provide recommendations to the government on the basis of their review.

##### Dietary Guidelines Advisory Committee

Prior to developing an updated edition of the *Dietary Guidelines for Americans* every 5 years, the USDA and HHS convene a Dietary Guidelines Advisory Committee (the Committee). This Committee consists of approximately 20 members, external to the Federal government, with expertise related to the topics and questions to be addressed. Members are nominated by the public, serve for up to 2 years without pay, and provide the USDA and HHS with independent, science-based advice and recommendations on a broad range of important diet and public health related topics. Committee members must complete the U.S. Office of Government Ethics Form 450 Confidential Financial Disclosure Report (19) prior to appointment and periodically throughout their term. All candidates’ financial disclosures are reviewed by the USDA or HHS Office of Ethics to ensure candidates have no financial, ethical, legal, and/or criminal COI that would prohibit serving on the Committee. Members are appointed as Special Government Employees and must comply with applicable COI statutes, regulations issued by the U.S. Office of Government Ethics, supplemental agency requirements, and other applicable Federal ethics rules. Members receive ethics training on these rules and regulations prior to beginning service on the Committee. A FAC is governed under the Federal Advisory Committee Act (FACA); thus, members undergo training for the FACA and the processes used for reviewing the evidence. The Committee collaborates with Federal staff, including NESR, to conduct systematic reviews, data analyses, and food pattern modeling on diet and public health-related topics and questions. Evidence from systematic reviews, data analyses, food pattern modeling, and existing reports is then integrated to develop a Scientific Advisory Report (20) that includes independent, science-based advice and recommendations for HHS and the USDA. The Departments consider this report which, along with input from Federal agencies and the public, informs the development of the next edition of the *Dietary Guidelines for Americans* issued jointly by the Secretaries of USDA and HHS (21).

#### Public stakeholders

Public stakeholders encompass a broad range of non-Federal individuals and organizations who have an interest in NESR systematic reviews, the evidence supporting them, and/or the decisions that will be made based on the findings. Public stakeholders include individuals and organizations acting on their own behalf, such as consumers, health professionals, researchers, industry professionals, members of the media, students, academics, community partners, scientific societies, and others with interests in diet and health. Public stakeholders may have advanced degrees in nutritional science or a related field, subject matter expertise in topics related to diet and health, knowledge of the operations and needs of nutrition-or public-health related policies and programs, involvement with nutrition-related communications or education, lived experience with individuals and communities, or oversight of research funding. All public stakeholders who provide written or verbal comments are requested to disclose their affiliation.

## NESR’s systematic review process

### Initiating, funding, and managing NESR systematic review projects

Systematic reviews are time and resource intensive projects conducted in accordance with NESR methodology which describes the complex, multi-step process, and involves numerous collaborators. NESR systematic review projects address high-priority public health nutrition topics that reflect what decision makers need to make evidence-based policy and program decisions. Federal stakeholders are responsible for defining the project scope by identifying and describing high-priority topics that represent gaps in Federal program knowledge or dietary guidance, and for commissioning NESR to initiate systematic review projects to address these topics. NESR systematic review projects are funded solely by Federal government agencies and programs, based on the needs identified by Federal stakeholders and/or in support of the development of the *Dietary Guidelines for Americans*. Funding for NESR projects come from Federal agencies’ appropriated funds.

The NESR team of analysts and librarians are responsible for designing, managing, conducting, and documenting systematic review projects within established timelines and budgets. NESR identifies the types of expertise needed throughout the process and collaborates with Federal stakeholders to solicit input from experts and other stakeholders, as appropriate. NESR convenes TECs with minimal perceived or actual COI for many reviews; however, for Dietary Guidelines Advisory Committee systematic reviews, a team of designated Federal stakeholders is responsible for convening the Committee. NESR facilitates the systematic review project to incorporate input of each expert group member, who each bring a unique perspective to the review and to identify and manage potential intellectual conflict of interest during the review process.

### Conducting NESR systematic reviews

Each NESR systematic review project is designed to answer one or more nutrition questions of public health importance. NESR systematic reviews are conducted using systematic, transparent, rigorous, and protocol-driven methods to search for, evaluate, synthesize, and grade the strength of the eligible body of evidence. NESR’s methodology is carefully designed to reflect the delineation of roles between the NESR team and its collaborators, and to ensure a collaborative approach that promotes objectivity and minimizes bias and COI throughout the review process.

In general, Federal stakeholders identify the need for a systematic review project and provide early input for research protocol that specifies the rationale for the project and describes the context of decisions to be informed by the results of the systematic reviews. NESR coordinates, facilitates, and documents the work necessary to produce systematic reviews in accordance with NESR systematic review protocol. The expert group is responsible for developing the systematic review protocol or verifying protocols developed by previous expert groups, and for synthesizing the evidence to develop conclusion statements and grade the strength of the evidence – based on their content and methodological expertise. Having an external, expert group complete these parts of the process enhances the trustworthiness of the review, and reduces the perception of bias or COI. Trained and qualified NESR analysts and librarians work to support the expert group by objectively executing their protocol and completing the most time and resource intensive steps, including searching for and screening studies, extracting data, and conducting risk of bias assessments. In this way, the expert group has adequate time to review and evaluate the evidence and is fully responsible for the results of their systematic reviews. NESR ensures that the work is accomplished in a timely manner according to the established methodology to minimize and/or manage potential COI, and the expert group’s time and expertise is preserved for synthesizing evidence.

**Figure 2:**
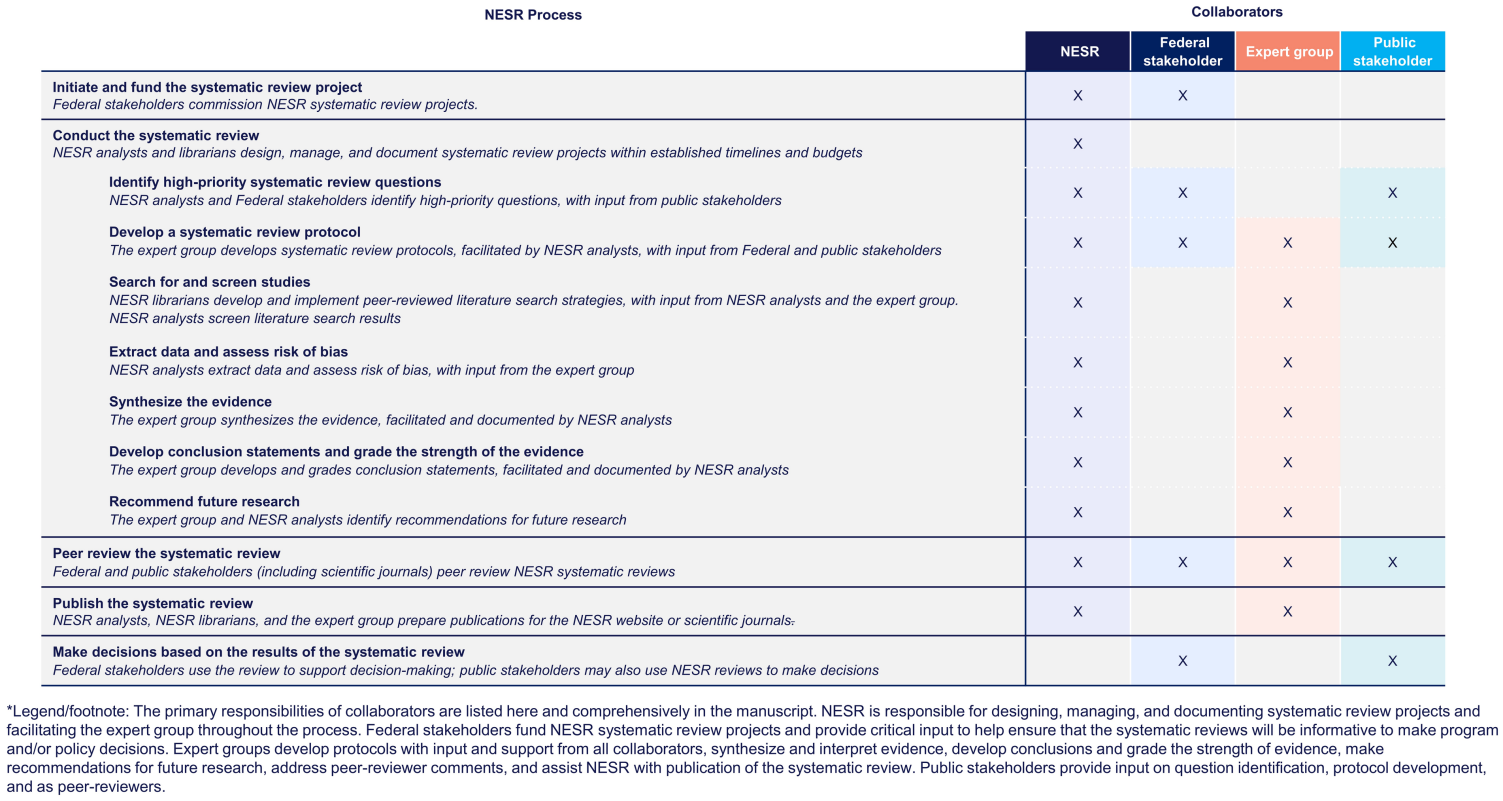
Systematic reviews conducted by the Nutritin Evidence Systematic Review team leverage input and support from various collaborators*.

Below is an overview of NESR’s systematic review methodology, which is described in greater detail in numerous reports and publications (21–24). This overview, along with Figure 2, provides a brief description of the major steps of NESR’s systematic review process and describes the roles and responsibilities that the NESR team and its collaborators play in implementing this methodology.

#### Identify high-priority systematic review questions

High-priority systematic review questions are identified, refined, and prioritized by NESR analysts and Federal stakeholders, considering input from public stakeholders.

Identifying the highest priority systematic review questions needed to address a topic requires consideration of the following factors:

- Relevance to and potential impact on Federal nutrition programs, policies, and consumer education priorities, including the *Dietary Guidelines for Americans,*
- Importance to public health, and
- Desire to avoid duplication of Federal efforts.

Federal stakeholders are responsible for identifying high-priority topics that represent gaps in Federal program knowledge or dietary guidance, and for commissioning NESR to initiate systematic review projects to address the topic. For some projects, NESR leads the process to identify high-priority systematic review questions (17, 24, 25), whereas in others, specifically Dietary Guidelines Advisory Committee projects, NESR supports the designated team of Federal stakeholders who lead question identification. NESR participation ensures that questions are within scope and can be answered using NESR systematic review methodology, and that the rationale for each review question is clear. NESR may support question identification, refinement, and prioritization by conducting evidence scans to determine whether sufficient evidence is available to conduct a review, or to identify existing work to use or build on. Public stakeholders also participate by providing input during question identification *via* a web-based public comments database, that Federal stakeholders consider when determining which questions will be examined to inform the *Dietary Guidelines for Americans*.

#### Develop a systematic review protocol

Systematic review protocols are developed by an expert group, with facilitation by NESR analysts, considering input from Federal and public stakeholders. All NESR systematic review protocols are posted online for transparency.

A systematic review protocol is a plan for how the specific systematic review will be conducted, and it is developed up front, before any evidence is reviewed. The systematic review protocol is designed to capture the most appropriate, relevant, and direct body of evidence to answer each question, and the strengths and limitations of various methodological approaches relevant to the questions are discussed. These upfront discussions and decisions are important because they inform all subsequent steps of the systematic review process. For the Dietary Guideline Advisory Committee, protocols are discussed during periodic public meetings. The public have an opportunity to provide comments on these protocols throughout the process *via* a web-based public comments database, as well as verbally during designated public meetings. These comments are taken into consideration by the expert group as they finalize their protocols and throughout the review process.

Each systematic review protocol includes an analytic framework, synthesis plan, and inclusion and exclusion criteria:

- The analytic framework defines the systematic review question (PICO: Population, Intervention/exposure, Comparator, and Outcome of interest), includes definitions for key terms, identifies key confounders and other factors that could affect the relationships examined, and helps ensure that important contributing elements in the causal chain will be examined and evaluated.
- The synthesis plan specifies how the evidence will be explored, synthesized, and organized in the systematic review based on each question of interest. In addition, the synthesis plan specifies the type of approaches to be used (e.g., synthesis with or without meta-analysis) and confirms visualization methods that will inform the synthesis (e.g., evidence tables).
- Inclusion and exclusion criteria specify characteristics of studies that lead to inclusion or exclusion from the body of evidence used to answer each systematic review question. Criteria are tailored to each systematic review question, and include study characteristics such as study design, publication date, population, country where research was conducted, intervention or exposure of interest, and outcome.

The expert group, facilitated by NESR analysts, develops and refines systematic review protocols that are appropriately tailored to ensure that the body of evidence is: applicable to the U.S. population, relevant to U.S. Federal public health nutrition policies and programs, and rigorous and strong from a scientific perspective. Expert groups may build upon a systematic review conducted by a previous expert group and verify the rigor and relevance of the existing protocols. NESR analysts may also solicit input from Federal stakeholders or conduct evidence scans to support protocol development and refinement to ensure the protocol is appropriate for its intended end-use. NESR analysts also monitor and manage potential COI during protocol development.

#### Search for and screen studies

Literature search strategies are developed, implemented, and documented by NESR librarians, with input from NESR analysts and the expert group. Search results are screened, and a manual search is conducted by NESR analysts. The goal of the literature search and screening process is to objectively identify the most complete and relevant body of evidence available to answer the systematic review question.

A literature search strategy identifies bibliographic databases, and constructs a search strategy optimized for each database (e.g., controlled vocabularies, field codes, search operators) that returns the complete body of relevant evidence while minimizing irrelevant results. NESR librarians develop, implement, and document a comprehensive literature search strategy using the expert group’s protocol. The expert group and NESR analysts may assist the NESR librarians in developing the search strategy by providing help on subject or topic terminology and technical terms to aid in choosing the most appropriate and comprehensive set of search terms possible. A second designated peer review librarian, who may be from NESR or from another Federal agency (e.g., National Institutes of Health/National Library of Medicine), provides comprehensive feedback on the search strategy’s accuracy, as well as usage of search operators, terms, subject headings, and databases. The peer review process provides additional rigor to the NESR process. NESR librarians use the feedback from the peer review librarian to finalize the search strategies and shares the revised search strategy with the expert group and NESR analyst (s) for final approval.

Next, the search is conducted. The result is a list of potentially relevant studies. Two NESR analysts independently and objectively screen the search results at three levels – title, abstract, and full text – excluding those that do not meet the criteria. After completing the dual screening, the analysts reconcile any conflicts identified. If necessary, a third analyst is consulted to resolve differences. The search results identify the most complete and relevant body of evidence available to answer the systematic review question. Studies that meet *all* criteria are included in the systematic review. NESR analysts also conduct a manual search to find peer-reviewed published studies not retrieved through electronic databases by hand searching reference sections of included studies and related systematic reviews and meta-analyses. The expert group may also help with manual searches by suggesting potential articles for which NESR should search reference sections. To provide transparency and reproducibility of the systematic review, NESR analysts support NESR librarians in documenting the entire literature search strategy and results according to PRISMA standards (26), including the full search strategy (including terms, operators, and date ranges) for each database, a complete list of articles that meet criteria for inclusion, and a list of excluded articles with the rationale for exclusion.

#### Extract data and assess risk of bias

NESR analysts extract data and assess risk of bias while considering decisions made by the expert group during protocol development.

A NESR analyst extracts and summarizes the data from each included study to objectively describe the body of evidence. Data relevant to the systematic review (e.g., study design, sample size, study setting, participant characteristics, interventions/exposures, outcomes, results, and funding source) is extracted using a standardized data extraction template. A second NESR analyst reviews the extracted data for completeness, accuracy, and consistent presentation and formatting. Any discrepancies noted by the second analyst are discussed and resolved, in consultation with a third NESR analyst and/or the expert group when needed. NESR analysts use the extracted data to create evidence tables that consistently and objectively present the key data from all studies included in the systematic review. Expert groups provide input on the types and format of data to be extracted and presented in evidence tables and figures.

Two NESR analysts independently complete risk of bias assessments for each included article using study-design specific tools (27–29). The analysts compare their responses, and any disagreements are discussed and reconciled. A third NESR analyst and/or expert group members may provide consultation to ensure consistency and accuracy of risk of bias assessments. This assessment evaluates how each included study was designed and conducted. The design and conduct of a study impact the extent to which its results are at risk of bias. Studies with lower risk of bias (i.e., with rigorous designs and sound analytic methods) are more likely to report accurate results. The risk of bias assessment is informed by expert group discussions and decisions made during development of the systematic review protocol. NESR analysts document the results of the risk of bias assessments.

#### Synthesize the evidence

Evidence is synthesized by the expert group, and the process is facilitated and documented by NESR analysts.

Evidence synthesis involves describing, comparing and contrasting, and combining all included studies to examine whether the intervention or exposure is related to the outcome in the population of interest. It aims to find overarching themes from the findings, identify and explain similarities and differences between studies, and determine whether certain factors may have affected the relationships being examined.

The expert group synthesizes the evidence according to the synthesis plan, which is developed as part of the protocol to help guide how the evidence would be prioritized, organized, synthesized, and presented in the systematic review. The expert group reviews and examines the body of evidence, considering study design, risk of bias assessments, characteristics of PICO elements, and key relationships between the intervention/exposure and outcome(s) of interest, looking for patterns of agreement and disagreement among the reported relationships. The expert group also determines whether methodological differences between the studies may help explain variations in effect sizes or degrees of association. NESR analysts facilitate discussions, monitor and manage potential COI, capture and integrate input from all members, resolve points of uncertainty, and document decisions of the expert group as they synthesize the evidence. NESR analysts also provide additional transparency to the expert group’s synthesis by drafting and/or refining visual presentations of the data.

#### Develop conclusion statements and grade the strength of the evidence

Conclusion statements are developed and the strength of evidence is graded by the expert group, a process facilitated and documented by NESR analysts.

Evidence synthesis results in development of a conclusion statement, which is one or more summary statements carefully constructed to answer the systematic review question. Conclusion statements reflect only the evidence reviewed and synthesized as outlined by the protocol and do not take evidence from other sources into consideration. Conclusion statements do not draw implications and should not be interpreted as dietary guidance.

The strength of evidence underlying each conclusion statement is graded to communicate the level of certainty in the conclusions (23). Grading involves evaluation of five elements (consistency, precision, risk of bias, directness, and generalizability) while taking study design into consideration, and results in the assignment of one of four grades (Strong, Moderate, Limited, and Grade Not Assignable).

The expert group develops conclusion statements, focusing on general agreement among studies, acknowledges areas of disagreement if and where they exist, and/or identifies relevant parameters when appropriate (e.g., evidence is applicable to only one sex). The expert group then assigns a grade to each conclusion statement using the NESR grading rubric to ensure that the final grade reflects consideration of all the grading elements. This grading process promotes consistency across systematic reviews, and allows the expert group to transparently document the strength of evidence underlying their conclusion statement. NESR analysts facilitate discussions to ensure compliance with NESR methodology, and document decisions of the expert group as they develop conclusion statements and grade the evidence underlying each conclusion statement. For reviews conducted with a Dietary Guideline Advisory Committee, the Committee describes their findings, synthesis, conclusion statements and grades during periodic public meetings and considers public comments throughout the review process. The public can attend public meetings to hear the Committee’s deliberation, and are able to submit comments on the Committee’s work through an web-based database. All public comments are collected by the agencies and shared with the Committee for careful consideration and incorporation into the process as feasible.

#### Recommend future research

Research recommendations are identified and documented by the expert group and NESR analysts.

The expert group and NESR analysts identify gaps and methodological limitations in the evidence throughout the review process. NESR analysts facilitate discussions and document decisions of the expert group as they recommend future research. Research recommendations describe the studies, data, and methodological advances that are needed to strengthen the body of evidence on a particular topic.

#### Peer review

NESR systematic reviews are peer-reviewed to ensure that the graded conclusion statements are supported by the evidence synthesized. The peer review described in this section is specific to the draft systematic review report; posting of the protocol and peer review of literature search strategies is described and discussed in previous sections. Different approaches to peer review are used depending on the project and may involve peer review coordinated by the journal to which a systematic review manuscript has been submitted, or it may involve peer review by Federal or non-Federal subject matter experts. During the 2020–2025 Dietary Guidelines, the peer review process was coordinated and managed by the USDA’s Agricultural Research Service. Regardless of the approach taken, peer reviewers are individuals who are external to, or not involved in, the systematic review project and who have either subject matter or methodological expertise. NESR analysts are responsible for submitting the draft systematic review for peer review. When peer reviewer comments are returned, the NESR team evaluates the comments and addresses any that are editorial in nature. Substantive comments are reviewed and discussed by the expert group. The NESR team documents revisions and responses, and provides them back to the peer reviewing entity, who then assesses the responses and ensures that the reviewer comments are adequately addressed.

#### Publish the systematic reviews

NESR systematic reviews are made accessible online (see text footnote 1, respectively). Each systematic review includes a plain language summary, a technical abstract, and a full systematic review containing complete documentation of each step of the review process. The systematic review also presents the literature search strategy, the list of articles that were excluded at the full-text level, and the funding for the systematic review. In some cases, a peer-reviewed publication is also prepared by NESR and the expert group.

NESR analyst and librarians are responsible for ensuring that the systematic review documents each step of the review process, including all substantive decisions made by the expert group. The NESR team also ensures that the report is in compliance with Federal agency directives by making sure the document is accessible to people with disabilities (30). The expert group is responsible for reviewing the final systematic review to ensure that it accurately captures their decisions.

### Make decisions based on the results of the systematic reviews

The main output of a NESR systematic review is one or more graded conclusion statements that document the current state of science. When NESR works with a TEC, the input of the TEC stops with the graded conclusion statement (s). And, because a TEC is not a FAC, it does not draw implications from the review or integrate findings from multiple reviews to provide recommendations to the government on the basis of their review. The TEC’s individual completed systematic reviews are provided to the Federal stakeholders who commissioned the work and are responsible for using the review to support decision-making, including the development of nutrition education materials, research agendas and funding priorities, and policy decisions.

When NESR works with a FAC, such as the Dietary Guidelines Advisory Committee, the Committee’s work extends beyond that of a TEC. As a FAC, the Committee is tasked with not only reviewing the science, but also integrating the findings from multiple approaches (data analyses, food pattern modeling, systematic reviews, existing reviews conducted by a TEC) and drawing implications based on that integration in the Scientific Advisory Report. This report includes independent, science-based advice and recommendations for HHS and the USDA and is *not* the next edition of the *Dietary Guidelines for Americans*. The Departments consider this report which, along with input from Federal agencies and the public, informs the development of the next edition of the *Dietary Guidelines for Americans* issued jointly by the Secretaries of USDA and HHS.

Every NESR project is commissioned with the intent that it will be used by Federal stakeholders to inform evidence-based policy and program decisions. It is rare that a single NESR systematic review is used to make a decision; rather, decisions are often informed by an integration of evidence from multiple NESR systematic reviews and other sources of information.

Table 1 illustrates two examples of how NESR systematic reviews are used to make decisions – one review conducted by a TEC, and the other conducted by the 2020 Dietary Guidelines Advisory Committee.

**Table 1:** Contrasting the roles and responsibilities of Technical Expert Collaboratives (TEC) and Dietary Guidelines Advisory Committees for conducting NESR systematic reviews: A case study.

	Pregnancy and birth to 24 months project	2020 Dietary guidelines advisory committee project
*Initiate, fund, and manage the systematic review project*		
Initiate the systematic review project	Federal stakeholders based on the Agricultural Act of 2014 (31)	Federal stakeholders, based on the National Nutrition Monitoring and Related Research Act of 1990 (32)
Fund the systematic review project	U.S. Department of Agriculture	U.S. Department of Agriculture and the Department of Health and Human Services
Manage the systematic review project	NESR analysts and librarians	NESR analysts and librarians and Federal support staff
*Conduct the systematic review*		
Identify high-priority systematic review questions	NESR analysts, Federal stakeholders, with expert input (16, 24, 25, 33)	Federal stakeholders, NESR analysts with public input
Develop a systematic review protocol	TEC, with support from NESR analysts and librarians	2020 Dietary Guidelines Advisory Committee (2020 Committee), with support from NESR analysts and librarians, with public stakeholder input
Search for and screen studies	NESR analysts and librarians	NESR analysts and librarians
Extract data and assess risk of bias	NESR analysts	NESR analysts
Synthesize the evidence	TEC	2020 Committee
Develop a conclusion statement and grade the strength of the evidence	TEC	2020 Committee
Recommend future research	TEC and NESR analysts	2020 Committee and NESR analysts
Peer review	Peer reviewers selected by the editors of the American Journal of Clinical Nutrition (24)	Federal stakeholders external to the project; and for 3 reviews, peer reviewers selected by the editors of the journal in which they were published (20, 34–36)
Publish the systematic reviews	NESR website and in a supplement of the American Journal of Clinical Nutrition (24, 37)	NESR website and 3 reviews were also published in peer-reviewed journals (34–36, 38)
*Make decisions based on the results of the systematic reviews*		
Integrate the evidence	N/A	2020 Committee developed their Scientific Report by integrating evidence from systematic reviews^a^, data analyses, and food pattern modeling (20)
Establish dietary guidance or make another Federal decision	N/A	Federal stakeholders from USDA and HHS developed the *Dietary Guidelines for Americans, 2020–2025* (21)

a The 2020 Committee conducted new systematic reviews, updated existing systematic reviews conducted by a previous Dietary Guidelines Advisory Committee or TEC, or used existing systematic reviews conducted by a previous TEC as-is without updating.

## Discussion

NESR has established itself as a key resource for the Federal government’s evidence-informed decisions related to public health nutrition, such as the development of the *Dietary Guidelines for Americans*. As an entity that specializes in conducting systematic reviews, NESR is unique in that every NESR review is commissioned with the specific intent of providing Federal stakeholders with information needed to make public health-related decisions. Given the impact that these decisions have on the health of Americans, NESR takes the rigor of its work seriously, producing systematic reviews that are high-quality, trustworthy, and useful to end users.

The NESR systematic review process, by design, leverages the input and support of its collaborators, including Federal and public stakeholders, as well as two different types of expert groups (TEC or Dietary Guidelines Advisory Committee). NESR utilizes collaborator expertise while maintaining division of responsibilities and managing potential biases and COI throughout the process. Federal stakeholders provide crucial input early in the process to define the scope of the systematic review project and the decisions it will inform. NESR works with expert groups, who develop the protocol, synthesize the evidence, draw conclusion statements, and grade the strength of the evidence. The NESR team supports these expert groups by objectively executing the protocol, completing resource intensive steps such as data extraction and risk of bias assessments, and documenting the systematic review. In this way, the expert group is fully responsible for the results of the systematic reviews. Given the expert group’s large scope of work and tight timeline, having a trained and qualified team like NESR to help with execution ensures that the work is accomplished in a timely manner. NESR supports the most time and resource intensive steps of the systematic review process, which helps to preserve the expert group’s time and expertise for later steps of the process.

There are no existing consensus best practices that specify who should play what roles in conducting systematic reviews, particularly those that are commissioned with the intent of informing guidelines or other types of decisions. A Committee of the National Academy of Sciences acknowledged,” it was not appropriate to prescribe a specific model by which systematic reviews are conducted” (39). This report emphasized the need for methodological experts with training and skills in evidence synthesis to conduct systematic reviews and encouraged an “ongoing and interactive relationship” between the systematic review team and the guideline developers to increase the validity and trustworthiness of the process. Further, they suggested those conducting the reviews *not* be completely isolated from those developing guidelines. However, the National Academies of Sciences, Engineering, and Medicine report, “Redesigning the Process for Establishing the Dietary Guidelines for Americans,” (3) called for a clear separation of roles between NESR and a “Dietary Guidelines Scientific Advisory Committee (DGSAC),” with NESR staff planning and conducting systematic reviews with input, primarily in developing protocols, from technical expert panels. The DGSAC, who would not be directly involved in conducting the reviews, would be responsible for interpreting and integrating the evidence from completed reviews. NESR’s collaborative approach described in this manuscript ensures that NESR’s rigorous methodology is consistently applied across reviews. NESR’s review of the evidence, including evidence synthesis, is the independent work of an external, expert group in accordance with the Federal Advisory Committee Act (40) – which enhances the review’s trustworthiness and reduces the perception of bias or COI. NESR’s approach also ensures that the Federal stakeholders, who are responsible for developing guidelines or making decisions based on the results of NESR’s reviews, provide input on the systematic review questions and protocol and retain some level of interaction with the NESR team and expert group throughout the process to ensure clarity of the scope and rationale for the review questions.

Across organizations that conduct systematic reviews, one area in which there is clear agreement is the need for both methodological and subject matter expertise of the team who conducts the systematic review (3, 39, 41–44). For example, the Cochrane Handbook requires that clinical and methodological expertise be represented among the systematic review author team. In addition, specific steps of the systematic review process call for certain types of expertise (41). NESR and other organizations involve information specialists, or librarians, in the development of literature searches. Librarian involvement correlates with higher quality reviews (45) and is critical for ensuring that searches are comprehensive and reproducible. Risk of bias assessments also require a review team with both content and methodological expertise (27). There is growing recognition that involving the public, such as patients or consumers, in the systematic review process is beneficial (46). This involvement takes many forms, with some groups posting protocols for public viewing (47), some groups, like NESR (38), post protocols for public comment (42, 43, 48), and some groups including patients or consumers on the systematic review team (46).

While there are a variety of collaborative approaches used by evidence synthesis organizations to conduct systematic reviews, there is consistent recognition about the need to manage potential or perceived COI among those involved in the review process. Perceived direct or indirect COI, such as intellectual, financial, or institutional, are commonplace in science (44). NESR takes steps to minimize and manage potential conflicts, with the goal of producing trusted reviews with minimal bias. These steps allow for input to be leveraged from a diverse group of collaborators in a way that does not introduce real or perceived bias (42, 49–51).

In general, NESR takes the view that within the field of systematic review, there is not a single, consensus, “gold standard” method that all organizations should seek to adopt or emulate. Rather, organizations who conduct systematic reviews require flexibility to adapt to the evolving field of systematic review science, with a focus on aligning with best practices. It is NESR’s view that “best practices” applies to the context in which an organization conducts its reviews. Thus, “best practices” are not a one-size-fits-all approach, but rather the development and use of methods that are rigorous, transparent, and minimize bias – and that are tailored to the specific purpose and context of the work. As indicated above, it is critical that a systematic review team with the appropriate content and methodological expertise be assembled and engaged at appropriate points of the process. And that steps be taken to prevent and manage potential COI. This article describes NESR’s process in sufficient detail such that it could be adopted or adapted by other organizations or governments based on their purposes and resources, but is not a formal recommendation to do so.

### Future directions

NESR plans to continue learning and collaborating with a broad range of systematic review organizations as it develops and advances its methodology, taking care to understand the rationale underlying various methodological approaches. Future advancements should focus on conducting research and evaluations that can inform the development of best practices for determining who should play each role in conducting systematic reviews, and how potential COI should be identified and managed. There is a need for more publicly available information from systematic review organizations detailing their operating procedures and specifying who completes specific tasks in their systematic review process, and evidence to understand whether different models of collaboration impact the quality or trustworthiness of the a review.

NESR’s systematic review process leverages a diversity of expertise and experience, while identifying and managing potential biases and COI. NESR systematic reviews relies on input from many different collaborators, whose expertise and involvement are critical for ensuring the rigor and utility of NESR’s work.

## Author contributions

JO, RR, NC, LE, MH, and JS are responsible for the design, writing, and final content of the paper. CB, EC, AF, JK, BK, JN, SS-M, AW, and ES provided feedback on the manuscript. All authors contributed to the article and approved the submitted version.

## Funding

Funding for NESR projects come from Federal agencies’ appropriated funds.

## Conflict of interest

The authors declare that the research was conducted in the absence of any commercial or financial relationships that could be construed as a potential conflict of interest.

## Publisher’s note

All claims expressed in this article are solely those of the authors and do not necessarily represent those of their affiliated organizations, or those of the publisher, the editors and the reviewers. Any product that may be evaluated in this article, or claim that may be made by its manufacturer, is not guaranteed or endorsed by the publisher.

## Footnotes

^1^ https://nesr.usda.gov

## Abbreviations

CNPP: Center for Nutrition Policy and Promotion
COI: Conflict of Interest
FAC: Federal Advisory Committee
FACA: Federal Advisory Committee Act
HHS: U.S. Department of Health and Human Services
NESR: Nutrition Evidence Systematic Review
TEC: Technical Expert Collaborative
The Committee: Dietary Guidelines Advisory Committee
2020 Committee: 2020 Dietary Guidelines Advisory Committee
USDA: U.S. Department of Agriculture
